# Association of Inflammatory Markers With Disease Progression and the Severity of COVID-19

**DOI:** 10.7759/cureus.54840

**Published:** 2024-02-24

**Authors:** Stuti V Patel, Jaya M Pathak, Radhay J Parikh, Karan J Pandya, Priyal B Kothari, Arushi Patel

**Affiliations:** 1 Department of General Medicine, Baroda Medical College, Vadodara, IND; 2 Department of General Medicine, GMERS (Gujarat Medical Education & Research Society) Medical College, Gotri, Vadodara, IND

**Keywords:** covid-19, d-dimer in covid, c-reactive protein, serum ferritin, lactate dehydrogenase (ldh), inflammatory markers

## Abstract

Introduction

In December 2019, there was a massive outbreak of viral pneumonia, which had a high case fatality rate. Genetic sequencing of the virus showed similarity with severe acute respiratory syndrome coronavirus (SARS-CoV). It was later named novel coronavirus 2019 while the disease it caused was given the nomenclature of COVID-19. This deadly pneumonia outbreak was declared a pandemic by the World Health Organization (WHO).

Aim

To derive the strength of the correlation between blood levels of various inflammatory markers with the severity of COVID-19 pneumonia in patients affected with novel coronavirus 2019.

Materials and methodology

A prospective study was conducted on 300 confirmed cases of COVID-19 infection from August 2020 to July 2021 in SSG Hospital, Vadodara. Diagnosis of patients as confirmed cases of COVID-19 infection was done according to the WHO interim guidance for COVID‐19. Their inflammatory markers were done for this study. All COVID-19-positive patients who had given negative consent for enrollment were excluded from the study. Patients were classified based on the severity of acute respiratory distress syndrome (ARDS). Comprehensive medical record information, encompassing biodata, clinical symptoms, comorbidities, and laboratory investigations, was systematically collected. Patients were given the standard treatment protocol as per guidelines. Patients were subjected to detailed investigations comprising complete blood counts and inflammatory markers like C-reactive protein (CRP), lactate dehydrogenase (LDH), serum ferritin, and D-dimer. Patients were further investigated by chest X-ray (posteroanterior view) or high-resolution computed tomography of the thorax.

Results

A total of 300 confirmed cases of COVID-19 infection were included in this study. Most of them were males (52%) with a mean age of 51 years and 48% were females with a mean age of 55 years. The majority of patients (40%) did not have ARDS, 23.3% of patients had mild, 16.7% of patients had moderate, and 20% of patients had severe ARDS. Higher CRP levels, serum ferritin, and serum D-dimer were significantly associated with the severity of COVID-19 infection as compared to those having no symptoms (p < 0.05). Increased levels were associated with severe clinical manifestations of COVID-19. The sensitivity of CRP is 69% and specificity is 100% as a diagnostic marker for COVID-19 pneumonia in terms of ARDS. The sensitivity of ferritin is 88% and specificity is 81% as a diagnostic marker for COVID-19 pneumonia in terms of ARDS. The sensitivity of D-dimer is 94% and specificity is 89% as a diagnostic marker for COVID-19 pneumonia in terms of ARDS. The sensitivity of LDH is 93% and specificity is 84% as a diagnostic marker for COVID-19 pneumonia in terms of ARDS.

Conclusions

Current evidence from our study showed that higher levels of inflammatory markers such as CRP, LDH, D-dimer, and ferritin are associated with the severity of COVID-19 in terms of ARDS and thus could be used as significant prognostic factors of the disease. These indicators might support clinical decisions to identify high fatality cases and poor diagnosis in the initial admission phase.

## Introduction

In the last 20 years, fatal pneumonia outbreaks have been attributed to severe acute respiratory syndrome (SARS) and Middle East respiratory syndrome (MERS) [1,2]. In December 2019, an unexplained cluster of pneumonia cases surfaced in Wuhan, China [3]. This epidemic exhibited genetic similarities to the SARS coronavirus [4]. The World Health Organization (WHO) declared COVID-19 a pandemic with an approximate mortality rate of 4.6%, leading to major global healthcare concerns [5]. The clinical spectrum of disease with the SARS-CoV-2 virus ranged from asymptomatic cases to fatal conditions [6,7]. A dysregulated immune response in the form of cytokine release syndrome has been recognized as a characteristic feature of COVID-19 pneumonia [8,9]. Postmortem studies of patients with COVID-19 pneumonia showed over-activation of T cells, especially T-helper cells subtype TH17 and CD8+ T cells, which resulted in immune-mediated lung injury resembling acute respiratory distress syndrome (ARDS) [10]. Extrapulmonary COVID-19 complications involve dysfunction in other organs such as the heart, kidneys, liver, fulminant sepsis, and coagulopathy [3,11-14].

The activation of the immune system serves as a double-edged sword: it plays a crucial role in defending the body against infectious agents but concurrently leads to the release of inflammatory mediators, including D-dimer, C-reactive protein (CRP), ferritin, interleukin-10 (IL-10), tumor necrosis factor-alpha (TNF-a), lactate dehydrogenase (LDH), and procalcitonin (PCT), whose elevated concentrations are strongly associated with unfavorable outcomes and the progression of the disease [15-17]. In a study conducted by Zhou et al., it was observed that deceased patients exhibited significantly elevated levels of inflammatory markers in comparison to survivors [11]. The amalgamation of these findings suggests that an overactive immune response, as evidenced by increased inflammatory markers, may be linked to the severity and outcomes of COVID-19. In our pursuit to enhance comprehension of the immunopathology of SARS-CoV-2 infection, we investigated the connection between inflammatory parameters and the severity as well as outcomes of COVID-19.

## Materials and methods

This study employs a combination of retrospective observational and prospective study methodologies. The sample size consists of a cohort of 300 patients diagnosed with COVID-19 infection, who underwent inflammatory marker assessments as part of the study. The study population includes all patients confirmed with COVID-19, adhering to the WHO interim guidance, and admitted to SSG Hospital, Vadodara. Identification of SARS-CoV-2 infection was achieved through throat swab samples tested using real-time reverse transcriptase-polymerase chain reaction assays at admission. Data collection occurred over the period from August 2020 to July 2021. Inclusion criteria encompassed all confirmed cases of COVID-19 infection, while exclusion criteria involved patients who tested positive for COVID-19 but declined consent for enrollment. Berlin Criteria for Acute Respiratory Distress Syndrome were used for diagnosis and classifying the severity of illness. Medical records, encompassing biodata, clinical information, comorbidities, and laboratory investigations, were obtained, and patients received treatment per the Ministry of Health and Family Welfare, India (MOHFW) guidelines.

The 2012 Berlin definition of ARDS delineates it as a sudden and widespread inflammatory lung injury that causes an elevation in pulmonary vascular permeability, an increase in lung weight, and the depletion of aerated lung tissue. This leads to hypoxemia and the presence of bilateral radiographic opacities, coupled with an augmented venous admixture, heightened physiological dead space, and diminished lung compliance. Critical components of the Berlin definition of ARDS encompass a rapid onset of lung injury occurring within one week or less, bilateral opacities not explained by pleural effusion, lung collapse, and lung nodules, which is identifiable through chest CT or chest radiograph, a PF ratio <300 mmHg with a minimum of 5 cm H20 positive end-expiratory pressure (or continuous positive airway pressure), and a condition not entirely explained by cardiac failure or fluid overload. Severity is categorized as mild (partial pressure of oxygen/fraction of inspired oxygen (PaO2/FiO2) = 200-300), moderate (PaO2/FiO2 = 100-200), and severe (PaO2/FiO2 < 100) [18].

Investigations for COVID-19 patients involved comprehensive assessments, such as complete blood count (CBC), renal function tests (RFT), D-dimer, serum ferritin, serum LDH, CRP, procalcitonin, prothrombin time/international normalized ratio (PT/INR), activated partial thromboplastin time (APTT), arterial blood gas (ABG), chest X-ray, and electrocardiogram. Serial monitoring of inflammatory markers, including D-dimer, serum ferritin, CRP, and LDH, was conducted every alternate day, alongside close monitoring of the patient's clinical condition.

Statistical analysis was conducted employing Statistical Package for the Social Sciences (SPSS) version 20 (IBM Corp., Armonk, NY) to examine the correlation between different inflammatory markers and the severity of COVID-19. The association between the severity of COVID-19 and inflammatory markers was assessed using the chi-square test, with statistical significance set at a p-value of less than 0.05.

## Results

**Figure 1:**
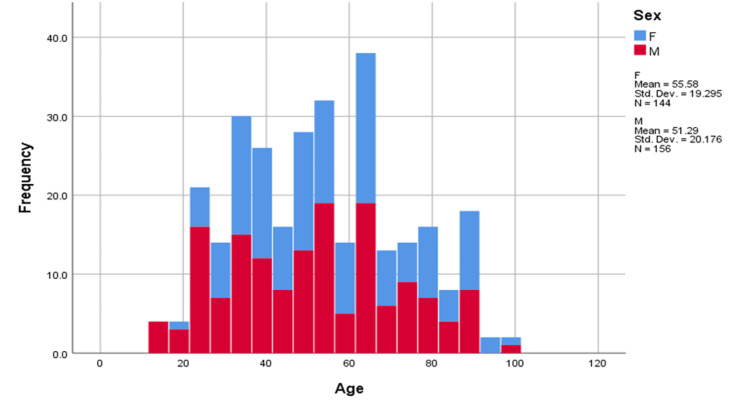
Age-wise distribution of male and female participants The figure above denotes the highest number of participants in the age group of 61-70 years, i.e., 51 patients, followed by 51-60 years, with 50 patients. The mean age of males was 51.29 + 20.176 years and that of females was 55.58 + 19.29 years. M = male; F = female.

A total of 300 confirmed cases of COVID-19 infection were included in this study. Of them, 52% were males (n = 156) with a mean age of 51 years and 48% were females (n = 144) with a mean age of 55 years (Figure 1).

**Figure 2:**
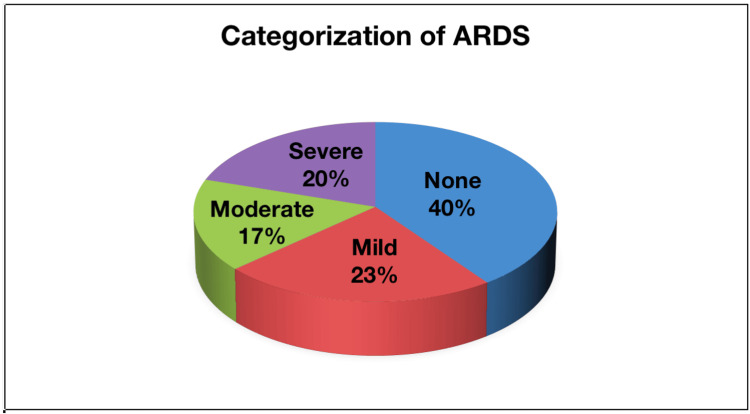
Classification of participants based on the categorization of ARDS Number of patients in each class of ARDS: mild (70/300 patients); moderate (50/300 patients); severe (60/300 patients); none (120/300 patients). ARDS: acute respiratory distress syndrome.

All the patients were assessed for the severity of COVID-19 according to the Berlin criteria. The majority of the participants, i.e., 120/300 (40%), did not have ARDS, 70/300 (23.3%) patients had mild, 50/300 (16.7%) patients had moderate, and 60/300 (20%) of patients had severe ARDS (Figure 2).

The outcome of interest in the current study was the association of various inflammatory markers with the severity of COVID-19 as understood by their categorization as suffering from mild, moderate, or severe ARDS, or no ARDS. Higher CRP levels were significantly associated with the severity of COVID-19 infection as compared to those having no symptoms (p < 0.05). Serum ferritin levels were associated with severe clinical manifestation of COVID-19 and the difference was statistically significant (p < 0.05). Serum D-dimer levels were significantly associated with severe clinical manifestation of COVID-19 (p < 0.05) (Table 1).

**Table 1:** Association of various inflammatory markers with the severity of COVID-19 ARDS: acute respiratory distress syndrome; CRP: C-reactive protein.

Inflammatory markers		Categorization of ARDS				Total (n = 300)	P-value (chi-square)
		None (n = 120)	Mild (n = 70)	Moderate (n = 50)	Severe (n = 60)		
CRP	<3	39	0	0	0	39	0.0001
	≥3	81	70	50	60	261	
Serum ferritin	Normal	100	11	0	13	124	0.0001
	High	20	59	50	47	176	
Serum D-dimer	<250 mg/L	109	1	1	12	123	0.0001
	≥250 mg/L	11	69	49	48	177	

**Figure 3:**
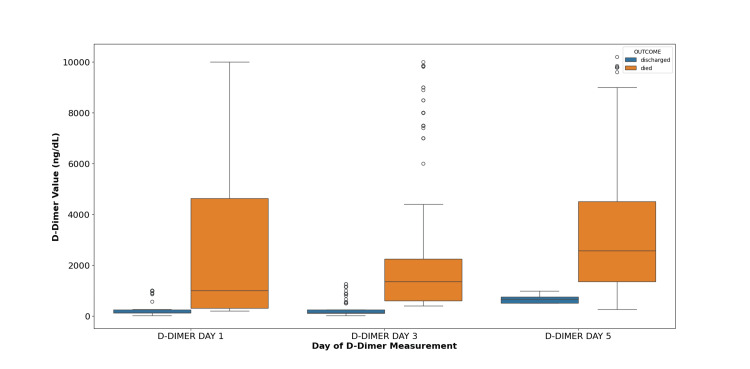
Mean D-dimer levels over follow-up for participants according to outcome The box whisker plot for D-dimer levels on days one, three, and five was obtained serially in participants with mild, moderate, and severe acute respiratory distress syndrome (ARDS) in COVID-19 pneumonia and their comparison in survivors and non-survivors groups has been shown above. The average values of D-dimer levels are significantly raised in non-survivors as compared to the survivors. Mean values on days one, three, and five and their standard error of the mean were as follows: Survivors: day one: 274.86 ± 25.02; day three: 285 ± 27.02; day five: 647.73 ± 10.37. Amongst those who died: day one: 2493.5 ± 254.53; day three: 2580.63 ± 237.29; day five: 3519.33 ± 223.38.

Participants who suffered from adverse outcomes (i.e., death) had a mean serum D-dimer level higher than the mean serum D-dimer levels of participants who were eventually discharged over the three days (days one, three, and five) of observation (Figure 3).

**Figure 4:**
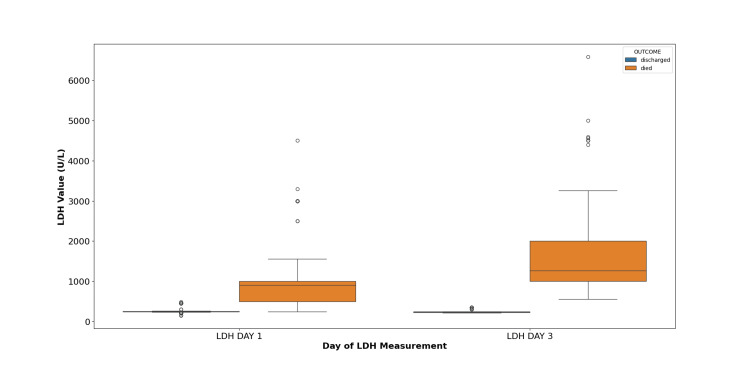
Mean lactate dehydrogenase (LDH) levels over follow-up for participants according to outcome The box whisker plot for LDH levels on days one and three was obtained serially in participants with mild, moderate, and severe acute respiratory distress syndrome (ARDS) in COVID-19 pneumonia and their comparison in survivors and non-survivors groups has been shown above. The average values of LDH levels are significantly raised in non-survivors as compared to the survivors. Mean values on days one and three and their standard error of the mean were as follows: Survivors: day one: 269.8 ± 6.60 and day three: 254.74 ± 4.25. Amongst those who died: day one: 1036.53 ± 64.57 and day three: 1677.2 ± 87.74.

The mean level of serum LDH decreased from day one to day three in the case of participants who were eventually discharged. However, for participants who eventually died, the serum LDH increased from day one to day three. This indicates that increasing serum LDH may be indicative of poorer prognosis in patients with COVID-19 (Figure 4).

## Discussion

In our study, among 300 patients, 156 were male, with a mean age of 51 years, and 144 were female, with a mean age of 55 years. Notably, the largest demographic group, constituting 17% (n = 51/300), fell within the age range of 61-70 years, while the smallest group, comprising 2.7% (n = 8), belonged to the age group of 20 years or below, which is consistent with findings observed in studies conducted in South Korea and China [19]. Prompt recognition of risk factors for severe illness is crucial for healthcare professionals to implement timely interventions and mitigate mortality rates. Elevated levels of inflammatory markers may manifest in individuals infected with various pathogens, including SARS-CoV-2. We have conducted a study to find out the association between putative serological markers of clinical severity and ARDS categorization (i.e., none, mild, moderate, and severe).

In a systematic review conducted by Gómez-Pastora et al., the role of serum ferritin in COVID-19 was investigated. The study reported that serum ferritin levels in patients with non-severe COVID-19 pneumonia were typically less than 400 ng/ml [20]. However, in patients with severe COVID-19 pneumonia, hyperferritinemia (ferritin level > 400 μg/L) was observed upon admission, with levels being up to 5.3 times higher in these severe cases than in non-severe cases. The results of this study are in congruence with these findings, revealing significantly elevated serum ferritin in patients with severe ARDS. Furthermore, the sensitivity of serum ferritin as a diagnostic marker in predicting the severity of COVID-19 infection was found to be 88%, with a specificity of 81%. Gómez-Pastora et al.'s study, focusing on serum ferritin levels in hospitalized patients with COVID-19 pneumonia, aimed to distinguish between survivors and those who succumbed to the disease. Their findings revealed that patients who died had exhibited serum ferritin levels up to four times higher than observed in discharged patients. Wu et al., in their investigation involving 201 confirmed COVID-19 cases, reported that higher serum ferritin was an independent risk factor associated with the development of ARDS [21]. In a study conducted by Chen et al., 63 out of 99 patients had hyperferritinemia [22]. Patients with severe COVID-19 pneumonia have elevated serum ferritin levels indicating cytokine storm, which is associated with a grave prognosis.

Increased LDH is one of the most common laboratory abnormalities in patients with COVID-19 pneumonia. Acute lung injury is highly associated with elevated levels of LDH [23]. In a retrospective examination of COVID-19 patients conducted by Zhang et al. [24], those with severe disease exhibited elevated LDH levels (342.8 U/L) in comparison to patients with mild disease (200.8 U/L). According to Li et al. [25], the heightened serum LDH persisted as an independent risk factor for the mortality associated with COVID-19. Henry et al. [26] reported a statistically significant higher level of LDH in terms of ICU vs. non-ICU (350.51 vs. 195.46; p < 0.001) patients and in non-survival patients vs. survival patients (351.51 vs. 166.91; p < 0.001). Our receiver operating characteristic analysis showed that the sensitivity and specificity of LDH are 93% and 84%, respectively, as diagnostic markers of the severity of COVID-19 infection. We observed that the mean level of serum LDH decreased from day one to day three in participants who were eventually discharged; however, for participants who eventually died, the serum LDH increased from day one to day three. Considering that LDH isozyme 3 is found in pulmonary parenchyma, thus in patients with severe COVID-19 ARDS, higher levels of LDH are found. Thus, the elevation of serum LDH levels may serve as an indicator of a poorer prognosis in COVID-19 patients.

CRP, an acute phase reactant, is significantly increased in COVID-19 patients, indicating a strong association with poor prognosis. It is produced by the liver in response to interleukin-6 stimulation. In our investigation, 87% of patients exhibited CRP values exceeding 3 mg/L, while only 13% had values below this threshold. CRP's diagnostic sensitivity for assessing the severity of COVID-19 infection is 69%, with a specificity of 100%. Traditionally, higher levels of CRP would be taken as a sign of bacterial rather than viral infection [27]. However, in COVID-19 pneumonia, very high CRP levels have been observed. The study conducted by Chen et al. reported that higher plasma CRP level indicates severe COVID-19 pneumonia and an extended duration of inpatient care [28]. Shang et al. conducted a multivariate logistic regression analysis, identifying CRP as an independent risk factor for severe COVID-19 [29]. Elevated CRP levels serve as indicators of the extent of tissue involvement and can assist in diagnosing potential complicating factors.

We have shown that in individuals diagnosed with COVID-19, an elevation in D-dimer levels upon admission is a common occurrence, and it correlates with both heightened disease severity and increased in-hospital mortality. The diagnostic efficacy of D-dimer is noteworthy, with a sensitivity of 94% and specificity of 89% for identifying COVID-19 pneumonia in terms of ARDS. Analyzing the mean D-dimer levels over three days (days one, three, and five) indicates that participants experiencing adverse outcomes, such as death, exhibited a significantly higher mean serum D-dimer level compared to those who were eventually discharged. These findings suggest a strong association of serum D-dimer levels with severity of illness. Supporting our findings, Huang et al. conducted a study involving 41 patients hospitalized with laboratory-confirmed COVID-19, revealing that D-dimer values were nearly five-fold higher in individuals with severe disease compared to those without (p = 0.004) [22]. Similarly, Zhou et al., in their study of 191 COVID-19 patients, observed that D-dimer values were almost nine-fold higher in patients who succumbed to the illness compared to those who survived (p < 0.001) [11]. These results collectively emphasize the significant role of abnormal coagulation function, particularly elevated D-dimer levels, in the progression of COVID-19.

Limitations

Most of the patients in our study (40%) have no signs and symptoms of ARDS. Hence, we need a much larger sample size, which represents the higher severity of ARDS for the better association between deranged inflammatory markers and prognosis. The impact of other comorbidities in COVID-19 patients and their influence on inflammatory markers needs another study. The ethnicity of the sample was limited to the Indian subcontinent only. ​​​​​​​This study has not included patients who were isolated at home or were admitted at primary or community health centers. ​​​​​​​Serial monitoring of laboratory parameters was done on an inpatient basis only, as outpatient follow-up of patients was not part of the study.

## Conclusions

Current evidence from our study showed that higher levels of inflammatory markers, such as CRP, LDH, D-dimer, and ferritin, are associated with the severity of COVID-19 in terms of ARDS and thus could be used as significant prognostic factors of the disease. According to our study, the most sensitive marker that correlated with ARDS is D-dimer and the most specific was CRP. These indicators might support clinical decisions to identify high fatality cases and poor prognosis in the initial admission phase.
